# Performance of the LACE index to identify elderly patients at high risk for hospital readmission in Singapore

**DOI:** 10.1097/MD.0000000000006728

**Published:** 2017-05-12

**Authors:** Lian Leng Low, Nan Liu, Marcus Eng Hock Ong, Eileen Yining Ng, Andrew Fu Wah Ho, Julian Thumboo, Kheng Hock Lee

**Affiliations:** a Department of Family Medicine and Continuing Care, Singapore General Hospital; b Family Medicine Program, Duke-NUS Medical School; c Health Services Research Centre, Singapore Health Services; d Centre for Quantitative Medicine, Duke-NUS Medical School; e Department of Emergency Medicine, Singapore General Hospital; f Health Services and Systems Research, Duke-NUS Medical School; g School of Physical and Mathematical Sciences, Nanyang Technological University; h Singhealth Emergency Medicine Residency Programme, Singapore Health Services; i Department of Rheumatology and Immunology, Singapore General Hospital, Singapore.

**Keywords:** elderly, LACE, prediction, readmission, Singapore

## Abstract

Unplanned readmissions may be avoided by accurate risk prediction and appropriate resources could be allocated to high risk patients. The Length of stay, Acuity of admission, Charlson comorbidity index, Emergency department visits in past six months (LACE) index was developed to predict hospital readmissions in Canada. In this study, we assessed the performance of the LACE index in a Singaporean cohort by identifying elderly patients at high risk of 30-day readmissions. We further investigated the use of additional risk factors in improving readmission prediction performance.

Data were extracted from the hospital's electronic health records (EHR) for all elderly patients ≥ 65 years, with alive-discharge episodes from Singapore General Hospital in 2014. In addition to LACE, we also collected patients’ data during the index admission, including demographics, medical history, laboratory results, and previous medical utilization.

Among the 17,006 patients analyzed, 2051 or 12.1% of them were observed 30-day readmissions. The final predictive model was better than the LACE index in terms of discriminative ability; c-statistic of LACE index and final logistic regression model was 0.595 and 0.628, respectively.

The LACE index had poor discriminative ability in identifying elderly patients at high risk of 30-day readmission, even if it was augmented with additional risk factors. Further studies should be conducted to discover additional factors that may enable more accurate and timely identification of patients at elevated risk of readmissions, so that necessary preventive actions can be taken.

## 1 Introduction

By 2050, Asia will become the oldest region, with an estimated elderly population of 1 billion.^[1]^ Singapore, a racially and culturally diverse nation, faces the challenge of an aging population due to low local birth rates and increasing life expectancy. Singapore has one of the most rapidly ageing population in Asia and a projected 1 million or 20% of the country's population will be elderly by 2030.^[2]^ In 2010, the 30-day hospital readmission rate of patients aged above 65 in Singapore was 19.0%, which is higher than the overall all-cause readmission rate of 11.0%,^[3]^ a figure comparable to that in the United States.^[4]^ To the healthcare system, readmissions present a strain to limited healthcare resources and lead to increased healthcare costs,^[4]^ at the same time exposing the patient to hospitalization-related complications.

One strategy to reduce unplanned readmissions is the use of risk stratification to identify patients who are likely to be readmitted so that preventive measures can be developed. As an example, appropriate resources can be allocated to these patients to enhance the discharge planning process^[5]^ or post-discharge follow-up.^[6–8]^ Several predictive models for readmissions have been established^[9]^ in various countries, for example, PARR-30^[10]^ by the United Kingdom National Health System and the Length of stay, Acuity of admission, Charlson comorbidity index, Emergency department visits in past six months (LACE) index (calculated from 4 items, namely Length of stay, Acuity of admission, Charlson comorbidity index, Emergency department [ED] visits in past 6 months) derived in Ontario, Canada.^[11]^ These models were developed from health systems with unique socio-demographic characteristics and thus have unknown generalizability to other health systems. Furthermore, some models might have limited clinical utility due to the complexity of the model.^[12]^ One model that is simple to use is the LACE index, with potential clinical utility in Singapore due to its simple variables which can be easily obtained from electronic health records.^[13]^ Tan et al^[14]^ found that patients in Singapore with a LACE score ≥ 10 had an almost 5 times higher risk of 30-day unplanned readmission after index discharge. However, the LACE index was developed from middle-aged patients and was not validated for elderly patients. The performance of LACE index was poor in older UK and Danish populations, which raised questions over its clinical utility in Singapore's elderly population.

In this study, the primary aim was to validate the LACE index in an older Singapore population and the secondary aim was to improve the predictive performance of LACE index by adding in additional risk factors in a logistic regression model.

## 2 Methods

### 2.1 Study setting

We conducted a retrospective study at Singapore General Hospital (SGH). Singapore has a mixed healthcare system,^[15]^ which is funded through a system of compulsory savings, subsidies and price controls.^[16]^ The government provides partial subsidies for inpatient bills with copayment from the patient determined by his or her financial status. Each inpatient admission belongs to 1 of 4 ward classes of decreasing copayment requirement: A, B1, B2, and C, which is determined at the point of admission. This study was approved by Singapore Health Services’ Centralized Institutional Review Board where patient consent was waived.

### 2.2 Study population

We included patients aged ≥ 65 years with discharge-alive episodes from across all clinical specialties in SGH between January 2014 and December 2014. We excluded patients who died during index admission, non-Singapore residents, and those who had a discharge destination other than home at index discharge from the analysis. The first admission in 2014 is defined as the index admission and we counted no more than 1 readmission for each patient discharged within the same period. Non-Singapore residents represent foreigner patients who had sought treatment at a Singapore hospital. These patients were excluded as it is likely that they would return home after their treatment.

### 2.3 Data collection

Data were extracted from an administrative database of the hospital's electronic health records (EHR). In addition to the components of LACE index, other relevant variables were chosen a priori and according to literature.^[13,17,18]^ We selected only variables that are available early during admission so that the model can be clinically useful, and easily extracted from the hospital's EHR and are available to all hospitals in Singapore, which will enable potential external validation of our model. We identified comorbidities using International Classification of Diseases (ICD-10) codes^[19]^ in primary and secondary diagnoses 1 year before the index admission, based on which we calculated the Charlson comorbidity index (CCI). Other variables include patient demographics, clinical and laboratory results, prior utilization of healthcare system and socio-economic indicators such as rental status in public housing, admission ward class and mode of healthcare payment. In Singapore, 1 major indicator of socioeconomic status (SES) is the house ownership; also, staying in public rental house is a sensitive measure of SES.^[17,20]^ Residents in public rental housing belong to the lowest strata of SES.

Many psychiatric conditions such as depressive illnesses and psychoses are diagnosed and managed in the outpatient settings. Therefore, ICD codes may underestimate the true prevalence of these diseases. We used common psychiatric medications (Table 1) as proxy markers of depressive illnesses and psychoses to describe our cohort but excluded them as predictors as they were only available on discharge.

**Table 1:**
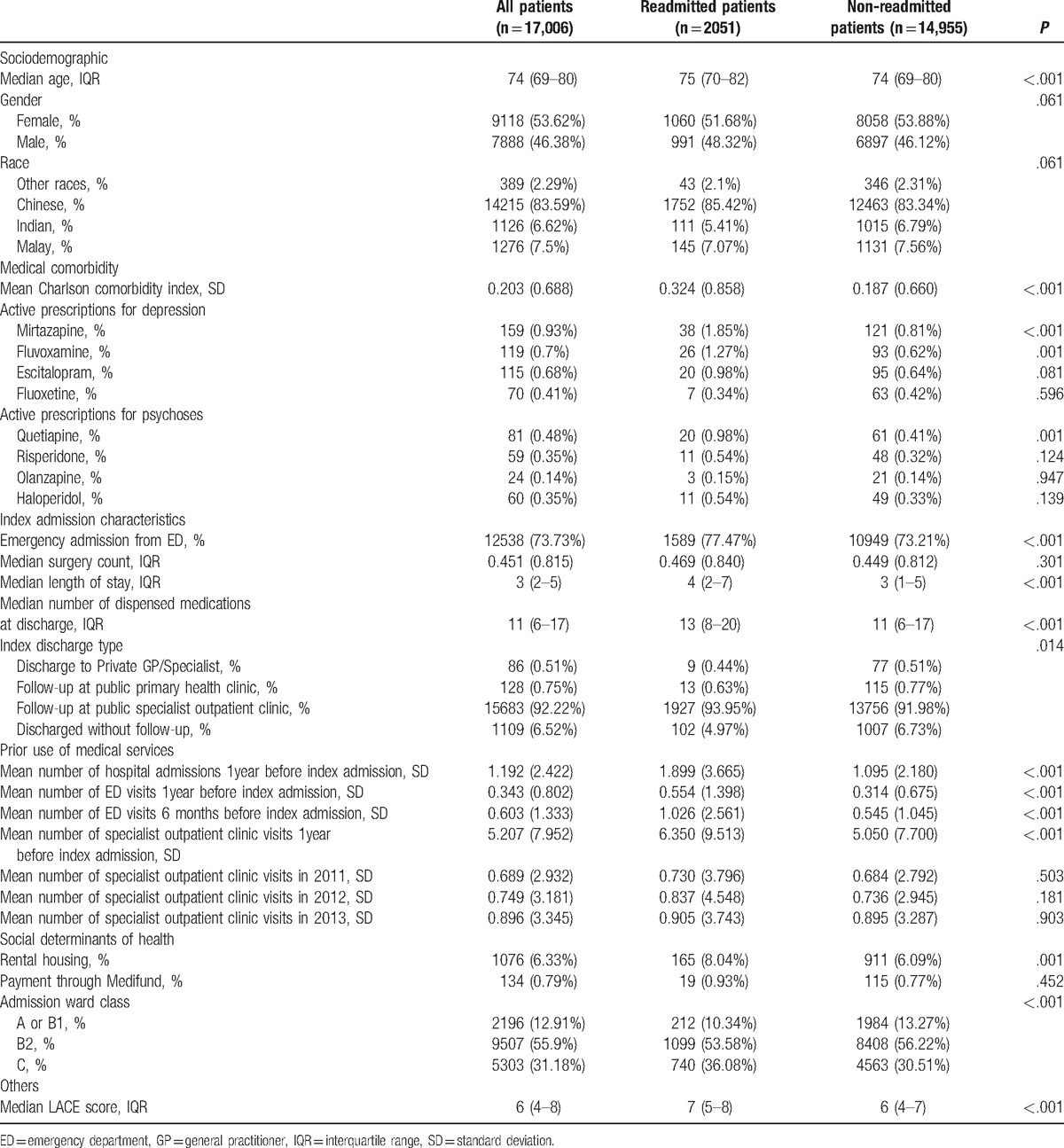
Description of the study cohort.

### 2.4 Statistical analysis

Baseline characteristics of the study population were analyzed by comparing patients with and without readmission to the hospital within 30 days. We used student's *t*-test or Wilcoxon rank-sum test to present means and standard deviations or medians and interquartile range of continuous variables. We used Chi-square test or Fisher's exact test to present categorical variables as numbers and percentage when appropriate. The statistical significance level was determined as *P* < .05. All variables were analyzed using 2-step logistic regression to study their predictive power in associating risk predictors with 30-day readmission. In the first step, the univariable logistic regression was applied to the entire set of variables, and in the second step, variables with *P* < .2 in step 1 were selected into multivariable logistic regression with stepwise variable selection. In the final regression model, in addition to statistically significant variables, clinically relevant predictors that were statistically nonsignificant were also included. The receiver operating characteristic (ROC) analysis was used as a diagnosis check to evaluate the predictive power. Data analyses in this study were performed using R version 3.2.3 (R Foundation, Vienna, Austria).

## 3 Results

**Figure 1:**
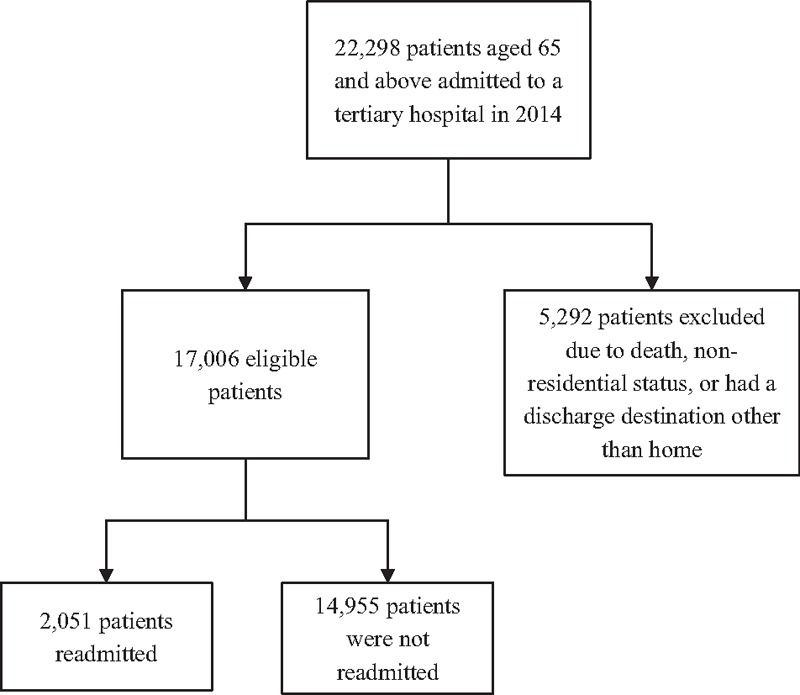
Patient selection criteria.

In total, 22,298 unique patients aged 65 and above were admitted to SGH in 2014. Among these, 5292 (23.8%) were excluded from analysis because of death during the index admission, nonresidential status, or who had a discharge destination other than home at index discharge (Fig. 1).

Of the 17,006 eligible patients, 2051 patients (12.1%) met the outcome, that is, 30-day readmissions after discharge. Characteristics of the study population (Table 1) show that the median age of the cohort was 74 (interquartile range [IQR] 69–80) years, the majority were female (53.6%, n = 9118), most admissions were emergent (73.7%, n = 12,538), the median length of stay was 3 days (IQR 2–5), and patients had a median LACE score of 6 (IQR 4–8). The readmitted patients were older as compared to the non-readmitted patients, had higher mean Charlson Comorbidity index, longer length of stay during the index admission, more dispensed medications at discharge, and higher LACE scores.

**Figure 2:**
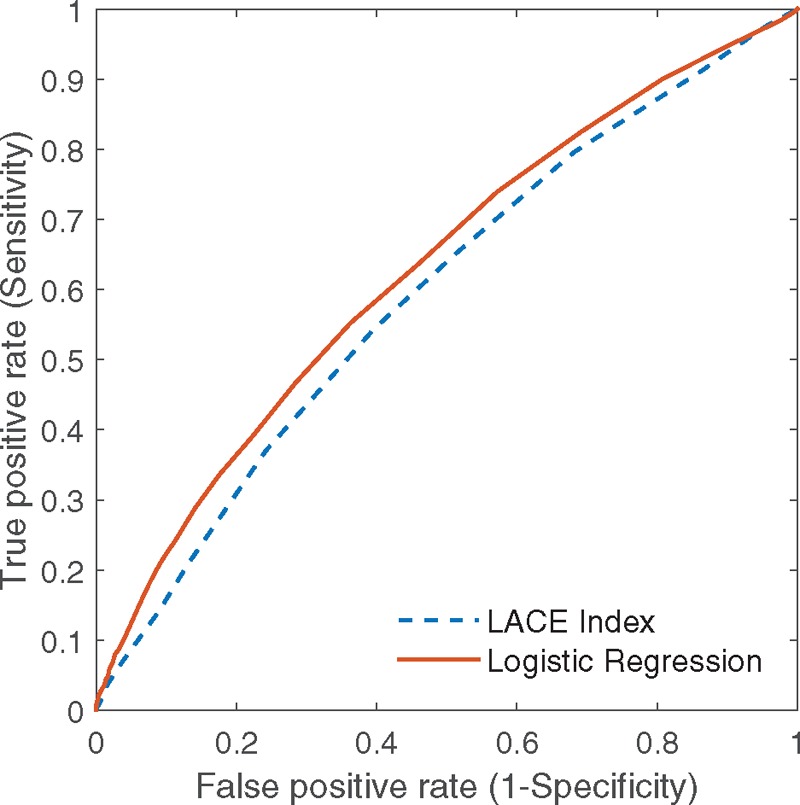
Receiver operating characteristic (ROC) curves of LACE index and logistic regression. LACE = length of stay, acuity of admission, charlson comorbidity index, emergency department visits in past six months, ROC = Receiver operating characteristic.

Table 2 shows the results of multivariable logistic regression where eleven variables were found highly predictive of 30-day readmission: Age, male gender, race, admission from ED, dispense count on index discharge, inpatient count 1 year before index admission, number of ED visits in previous 6 months, outpatient count in year 2012, rental housing, admission ward class B2 or C, and the LACE index. In the model, the LACE index has an odds ratio 1.089 and *P* < .001, which means that increment of 1 point in the LACE index will increase 8.9% odds for a patient to be readmitted within 30 days. The final logistic regression model achieved c-statistic of 0.628 (95% confidence interval [CI]: 0.615–0.642) (Fig. 2). As shown in Table 3, the optimal cutoff of the logistic regression model, which consists of LACE index with additional risk factors, provided greater sensitivity (55.1% vs 54.3%) as well as greater specificity (64.2% vs 60.4%) than the LACE index at the optimal cut-off score of 6.

**Table 2:**
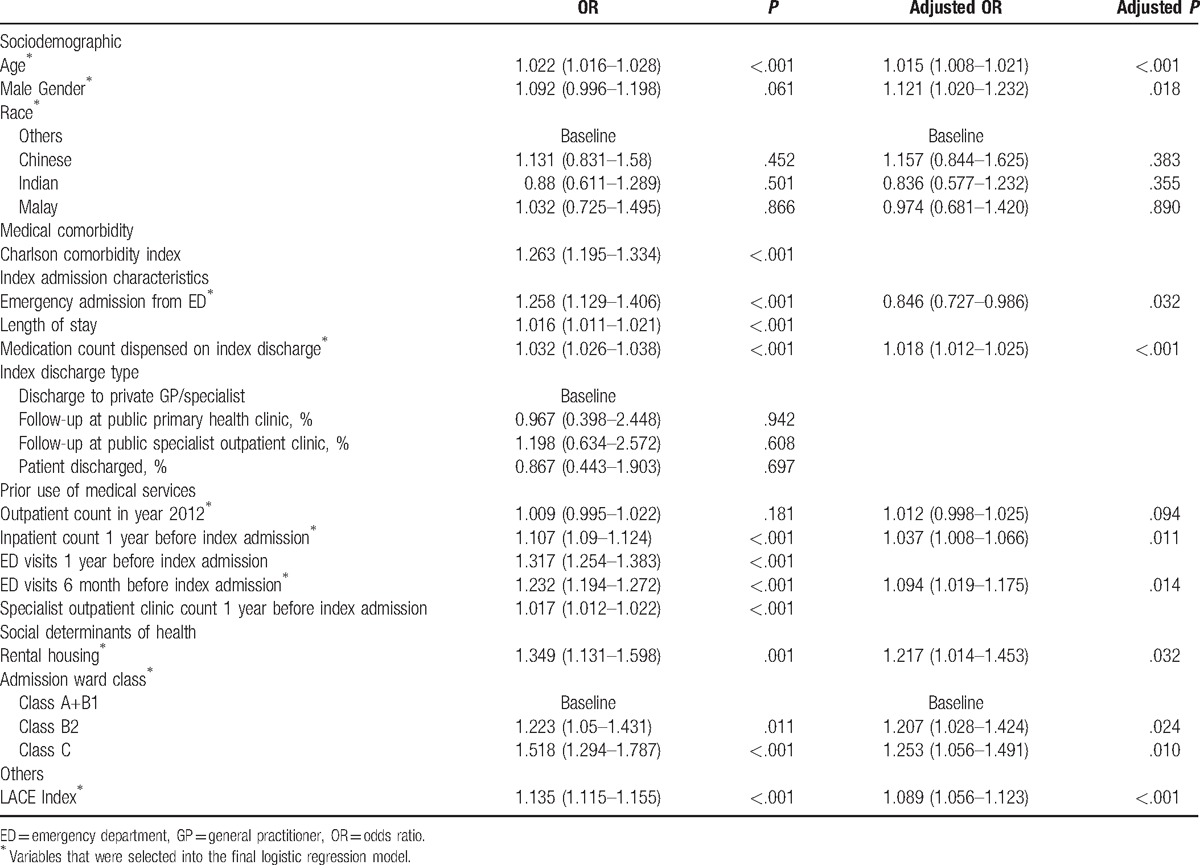
Univariable and multivariable logistic regressions.

**Table 3:**
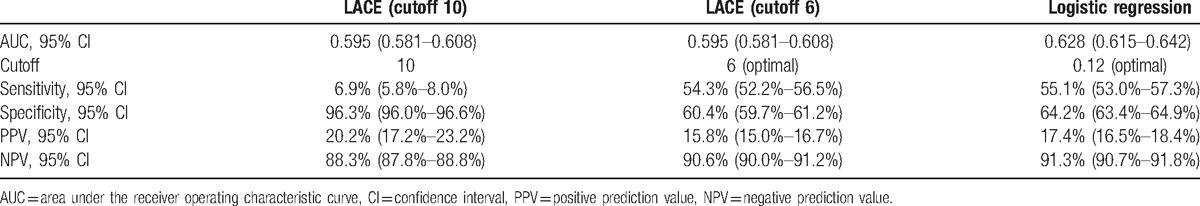
Discriminatory values of LACE and logistic regression model.

The LACE index alone achieved a c*-*statistic of 0.595 (95% CI: 0.581–0.608) and had poor discriminative ability. Analysis of the receiver operator characteristic (ROC) curve showed that in using the LACE index, cutoff score of 6 optimized the predictive performance when compared with cutoff score of 10. In our study cohort, the model with LACE cutoff of 6 correctly identified 9039 out of 14,955 patients as low risk of readmissions and who were not actually readmitted, and achieved a specificity of 60.4% (Table 3). The model incorrectly classified 937 patients as low risk of readmissions but were readmitted (false negatives), and 5916 patients were classified as high risk of readmission but was not readmitted (false positives). Furthermore, the model accurately predicted 1114 out of the 2051 readmitted patients with a sensitivity of 54.3%. This cutoff value of 6 provided greater sensitivity (54.3% vs 6.9%) with lower specificity (60.4% vs 96.3%) than the LACE index cutoff score of 10.

## 4 Discussion

This study aims to assess the performance of LACE index in identifying elderly patients at risk for readmission and to determine other risk factors that enhance the prediction of readmissions. In our population of patients aged 65 and above, the LACE index had poor discriminating ability at predicting readmissions in 30 days, as indicated by its low c-statistic of 0.595. This performance is poorer than when the LACE index was applied to a population of Singapore admissions of all ages in a previous study (c-statistic of 0.70).^[14]^ This is in agreement with the poor performance of LACE index among countries with older populations such as the UK and Danish population.^[21,22]^ The only component of the LACE index that is of statistical significance in predicting readmission risk is the number of emergency department visits in the previous 6 months. The poorer performance of the LACE index in our study cohort compared to the original derivation study could be attributed to the differences in age, which is one of the significant predictors for readmission risk that is consistent with the existing literature.^[23]^ The mean age of our study cohort was 75 years, whereas the mean age of the original derivative Canadian study population was 59 years.

Discriminative ability only improved slightly with the incorporation of additional variables to the LACE index. It could be possible that other predictors of readmission^[18]^ such as the level of social support,^[24]^ functional status,^[25]^ frailty,^[26]^ and patient activation levels^[27]^ are important but not available from our administrative database. Some better performing models incorporated a patient-completed survey of functional status in addition to administrative data^[23]^ but face practical difficulties as labor intensive data collection is required.

To our knowledge, we are the first to validate the LACE index in an older Singapore population. Since the LACE index, with or without additional risk variables, displayed poor to fair discriminative ability, it suggests much room for improvement of the model. Future studies should include more clinical, social, and functional variables, with costs and labor factors taken into consideration.

Singapore has started implementing a National Electronic Health Record (NEHR) since 2012 with plans for a cross linkage between hospitals’ EHR, NEHR, and other registries. With a larger pool of data, better prediction tools can be developed to identify high risk and high cost patients. Studies on various healthcare outcomes have shown that advanced machine learning techniques improve the performance of prediction models^[28]^ and should be considered as an alternative method for derivation of models.

There are limitations in our study. First, our prediction model was derived from administrative data, which may contain coding errors. Second, our selection of variables is confined to those which are routinely collected in the administrative database. Third, we were not able to substantiate deaths happened out of the study hospital and 30-day readmissions to hospitals other than SGH. However, previous studies have shown that majority of elderly die in hospitals in Singapore and only a minority utilized services from more than 1 hospital.^[29,30]^ In addition, patients are likely to return to SGH as it is the largest hospital in Singapore and also a national referral center. Fourth, caution ought to be exercised in generalizing our findings to other hospitals as some variables such as residence in public rental housing and ward payment class are unique to Singapore's healthcare system, and may need to be substituted with another surrogate of the patient's financial status if implemented in another healthcare system. Lastly, the preventability of readmission cases is unclear as our data did not provide the resolution to isolate cases that are deemed to be preventable.

## 5 Conclusions

In summary, the LACE index, with or without additional variables, had poor discriminative capability of stratifying elderly patients according to the risk of 30-day readmission in Singapore. Subsequent studies should be proposed to identify additional risk factors for the prediction of readmission risk to allow accurate and timely identification of a high-risk cohort for interventions that can prevent potentially avoidable readmissions.
